# Utilization of Diverse Molecules as Receptors by Cry Toxin and the Promiscuous Nature of Receptor-Binding Sites Which Accounts for the Diversity

**DOI:** 10.3390/biom14040425

**Published:** 2024-04-01

**Authors:** Ryoichi Sato

**Affiliations:** Graduate School of Bio-Application and Systems Engineering, Tokyo University of Agriculture and Technology, Naka 2-24-16, Koganei 184-8588, Tokyo, Japan; ryoichi@cc.tuat.ac.jp

**Keywords:** cry toxin, *Bacillus thuringiensis*, ATP-binding cassette transporter, cadherin-like receptor, receptor, resistance, susceptibility, low-efficiency receptor, promiscuous binding, directed evolution

## Abstract

By 2013, it had been shown that the genes cadherin-like receptor (Cad) and ATP-binding cassette transporter subfamily C2 (ABCC2) were responsible for insect resistance to several Cry1A toxins, acting as susceptibility-determining receptors, and many review articles have been published. Therefore, this review focuses on information about receptors and receptor-binding sites that have been revealed since 2014. Since 2014, studies have revealed that the receptors involved in determining susceptibility vary depending on the Cry toxin subfamily, and that binding affinity between Cry toxins and receptors plays a crucial role. Consequently, models have demonstrated that ABCC2, ABCC3, and Cad interact with Cry1Aa; ABCC2 and Cad with Cry1Ab and Cry1Ac; ABCC2 and ABCC3 with Cry1Fa; ABCB1 with Cry1Ba, Cry1Ia, Cry9Da, and Cry3Aa; and ABCA2 with Cry2Aa and Cry2Ba, primarily in the silkworm, *Bombyx mori*. Furthermore, since 2017, it has been suggested that the binding sites of BmCad and BmABCC2 on Cry1Aa toxin overlap in the loop region of domain II, indicating that Cry toxins use various molecules as receptors due to their ability to bind promiscuously in this region. Additionally, since 2017, several ABC transporters have been identified as low-efficiency receptors that poorly induce cell swelling in heterologously expressing cultured cells. In 2024, research suggested that multiple molecules from the ABC transporter subfamily, including ABCC1, ABCC2, ABCC3, ABCC4, ABCC10, and ABCC11, act as low-efficiency receptors for a single Cry toxin in the midgut of silkworm larvae. This observation led to the hypothesis that the presence of such low-efficiency receptors contributes to the evolution of Cry toxins towards the generation of highly functional receptors that determine the susceptibility of individual insects. Moreover, this evolutionary process is considered to offer valuable insights for the engineering of Cry toxins to overcome resistance and develop countermeasures against resistance.

## 1. Introduction

*Bacillus thuringiensis* is renowned for producing proteinaceous toxins, used globally for controlling insect pests in agriculture through commercial sprays and genetically modified insect-resistant crops (gene-modified *Bt* crops) [1]. These toxins encompass various protein families such as crystal toxin (Cry), cytolytic toxin (Cyt), and vegetable insecticidal protein (Vip), which were reclassified into 16 classes in 2021 [2]. Among these, Cry toxins are most frequently used due to their extensive research history, high insecticidal activity, and broad spectrum. However, resistance to Cry toxins has been emerging since the late 1980s [3], prompting investigations into the genetic basis of this resistance. In 2001, the cadherin-like receptor (Cad) gene of the tobacco budworm, *Heliothis virescens*, was identified through linkage mapping as a candidate gene for Cry1Ac resistance [4]. Similar findings were reported for the pink bollworm, *Pectinophora gossypiella*, and the cotton bollworm, *Helicoverpa armigera* [5,6]. Subsequently, in 2010, the ATP-binding cassette transporter subfamily C2 (ABCC2) was also linked to Cry1Ac resistance in the tobacco budworm, *H*. *virescens* [7], and similar associations were found in the diamondback moth, *Plutella xylostella*, cabbage looper, *Trichoplusia ni*, and beet armyworm, *Spodoptera exigua* [8,9]. Moreover, ABCC2-related resistance to Cry1Ab in silkworms was demonstrated, with mutations in ABCC2 shown to induce resistance through genome editing [10]. Resistance linked to the ABCC2 locus was also observed for Cry1Ac in *Helicoverpa armigera* [11] and for Cry1Fa in the fall armyworm, *S*. *frugiperda* [12,13,14], and the Asian corn borer, *Ostrinia furnacalis* [15].

Given that ABCC2 is a cell surface molecule, it was hypothesized early on to serve as a receptor for Cry1 toxin. This was empirically demonstrated using a heterologous expression system, where BmABCC2-expressing Sf9 cells showed swelling upon Cry1Aa administration, akin to the reaction in midgut columnar cells [16,17]. Similarly, *Drosophila melanogaster* larvae expressing PxABCC2 in the midgut succumbed upon consuming a diet with 0.05 ppm Cry1Ac [18], and *Drosophila melanogaster* wing disc cells expressing BmABCC2 swelled and died in response to Cry1Aa [19]. Additionally, the detection of apical-to-basal K^+^ flux by short-circuit current measurement in insect midguts treated with Cry1Aa and Ac suggested that Cry1A forms cation-permeable channels in lipid membranes [20]. The formation of such cation channels by Cry1Aa toxin was also confirmed in *Xenopus* oocytes expressing BmABCC2 [21], indicating that ABCC2 functions as a receptor facilitating the formation of cation channels by Cry toxins.

Before it was identified as a gene responsible for resistance, Cad was demonstrated to function as a receptor against Cry toxin. Specifically, when Cad of the silkworm, BmCad, was expressed in Sf9 cells and exposed to Cry1Aa, cell swelling was observed [22,23]. Moreover, the swelling of midgut cylindrical cells induced by Cry1Aa or Cry1Ac was inhibited by anti-BmCad (BmCadherin) antiserum [24]. However, the cell-swelling-inducing activity of BmCad in response to Cry1Aa in Sf9 cells was found to be ~5000 times lower than that of BmABCC2 [17]. In a two-electrode voltage clamp assay, where an equal number of cRNA molecules were injected into *Xenopus* oocytes, BmABCC2 displayed cation channel formation activity ~5000 times higher than that of BmCad [21]. According to the prevailing theory, resistance to Cry toxin in insects is thought to arise from mutations in high-functioning receptors. This raises the question of how both Cad and ABCC2 can be resistance factors simultaneously in the same insect [4,7]. The explanation provided through studies using the Sf9 cell heterologous expression system is that BmCad and BmABCC2 function cooperatively and synergistically to enhance the cell-swelling-inducing activity of Cry1Aa [17]. Moreover, when BmCad and BmABCC2 were co-expressed in *Xenopus* oocytes, the ion channel formation activity against Cry1Aa toxin was synergistically increased by ~8 times compared to when BmABCC2 alone was expressed [21]. These findings suggest that the synergistic activity of these two molecules explains why both Cad and ABCC2 can lead to resistance to Cry1 toxin [25]. Additionally, the synergistic operation of these molecules implies that Cad serves a distinct receptor function from ABCC2, possibly related to toxin gathering, as this synergy must arise from two types of molecules with different functions. Thus, Cry1Aa is posited to use two distinct receptor types collaboratively. However, the specific binding sites of Cry toxin on these receptors remained uncertain for some time.

**Figure 1:**
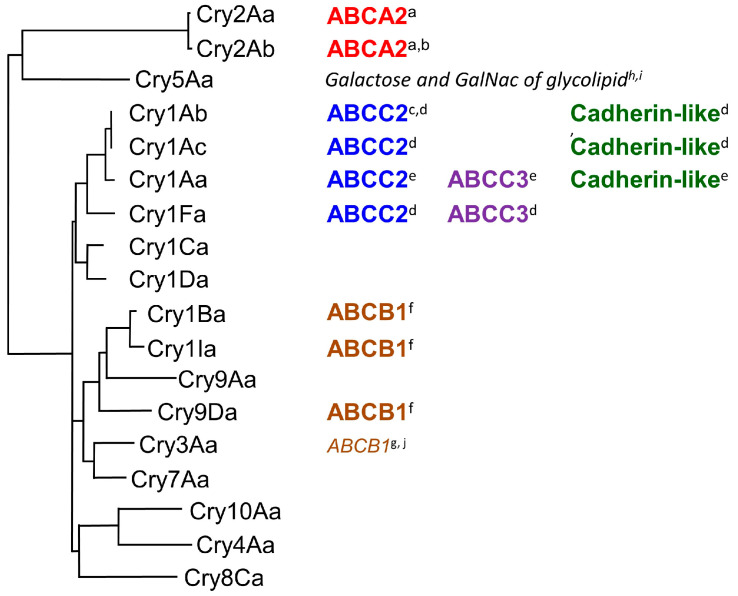
Molecules directly evidenced as susceptibility-determining receptors for Cry toxins. Multiple sequence alignment and phylogenetic tree construction of Cry toxins focused on the amino acid sequences of domain II, using CLUSTALW 2.1 at GenomNet (https://www.genome.jp/ accessed on 26 March 2024). Citations: a, [28]; b, [30]; c, [10]; d, [34]; e, [35]; f, [29]; g, [26]; h, [39]; i, [40]; j, [27].

**Figure 2:**
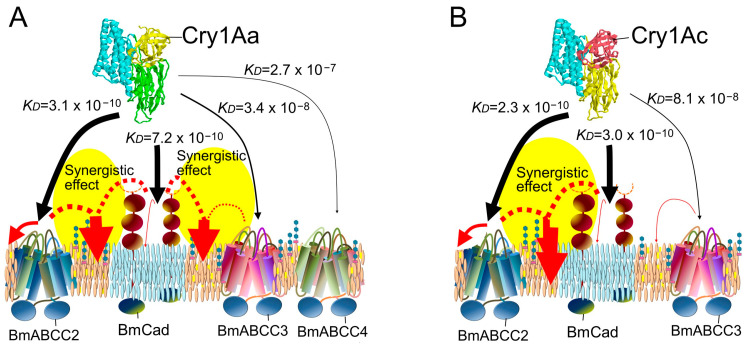
Models of susceptibility-determining receptors in the silkworm for Cry1Aa (**A**) and Cry1Ac (**B**), based on analyses of binding affinity between Cry toxins and receptors, cell-swelling-facilitating activity of receptors, and susceptibility-determining activity of receptors in silkworm larvae. Black arrows indicate the binding affinity of each subclass of Cry toxin to the receptors. Red arrows indicate cell-swelling-inducing activity of each toxin facilitated by a single receptor molecule or synergistic effects of two molecules.

**Figure 3:**
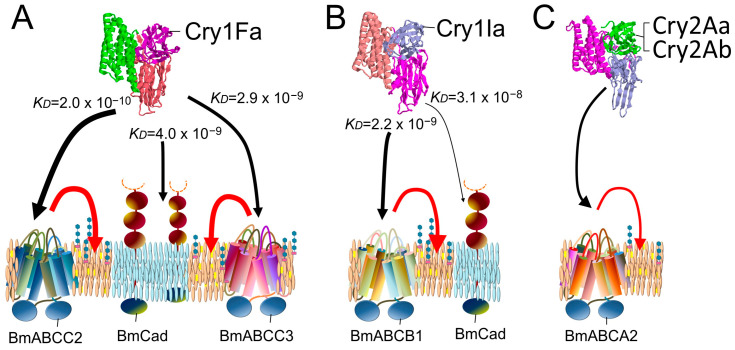
Models of susceptibility-determining receptors in the silkworm for Cry1Fa (**A**), Cry1Ia (**B**), and Cry2A (**C**) based on analyses of binding affinity between Cry toxins and receptors, cell-swelling-facilitating activity of receptors, and susceptibility-determining activity of receptors in silkworm larvae. Black arrows indicate the binding affinity of each subclass of Cry toxin to the receptors. Red arrows indicate cell-swelling-inducing activity of each toxin facilitated by a single ABC transporter molecule.

Since 2016, receptor functions for Cry toxins have been identified in ABC transporters of different subfamilies than ABCC2, including Cry2, Cry3, Cry1Ba, Cry1I, and Cry9Da [26,27,28,29,30] (Figure 1). Moreover, for Cry1A toxins, it has been suggested that not only ABCC2 but also ABCC3 and Cad in various combinations act as receptors determining the susceptibility of individual insects [9,31,32,33,34,35] (Figure 1). The receptor function of ABC transporters is also strongly influenced by their binding affinity for Cry toxin [25,36,37,38] (Table 1). Therefore, this review will first detail the molecules that determine susceptibility in insects as receptors, based on systematic analysis results since 2016 (Figure 2 and Figure 3).

**Figure 4:**
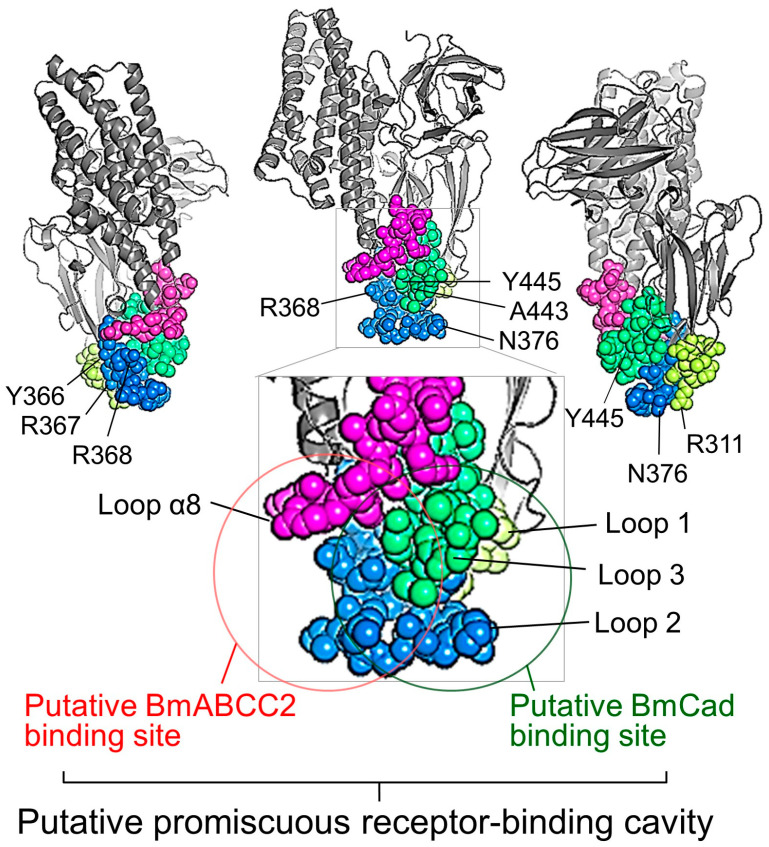
Putative promiscuous receptor-binding site on Cry1Aa. The putative promiscuous receptor-binding cavity, BmABCC2 binding site, and BmCad binding site are depicted using a space-filling model of PDB-1CIY (https://doi.org/10.2210/pdb1CIY/pdb, accessed on 26 March 2024), based on the report by Adegawa et al. (2019) [37].

As previously mentioned, evolutionarily distant Cry toxin subfamilies are known to use different ABC transporters as receptors. However, it has also been shown that Cry3Aa, Cry1Ia, 1Ba, and 9Da, despite being distantly related according to general phylogenetic analysis, use the same ABC transporter as a receptor [26,29] (Figure 1). This usage of identical receptors is attributed to similarities in domain II, the receptor-binding site of Cry toxins [29]. Domain II of Cry3Aa, Cry1Ia, 1Ba, and 9Da shares a close phylogenetic relationship, which explains their use of the same receptors. Furthermore, Cry1Aa is known to interact not only with ABC transporters but also with Cad as receptors. It has been reported that these receptors compete for binding to Cry1Aa, indicating a competitive relationship [43]. Accordingly, some information has been gathered about the binding sites for these two receptors on Cry1Aa toxin and its promiscuous binding characteristics [37]. Thus, as a second topic in this review, reports on the binding sites of Cry toxin to Cad and ABCC2 were summarized (Figure 4).

**Figure 5:**
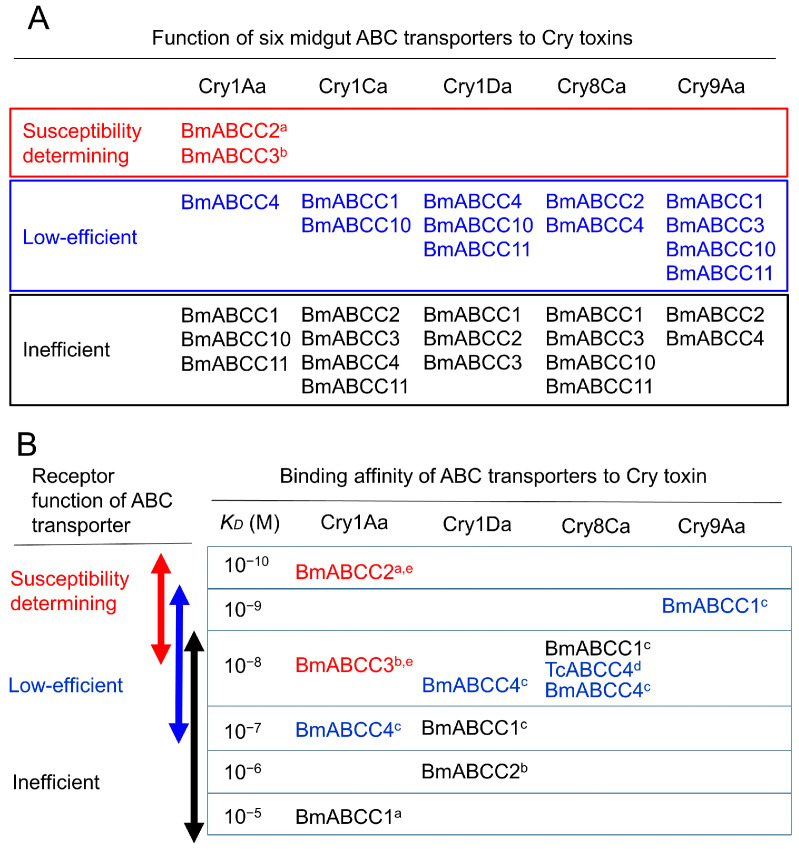
Relationship between binding affinity and function of ABC transporters to Cry toxins. (**A**) Functional classifications of ABCC molecules expressed in the *B*. *mori* midgut, cited from [41]. (**B**) Relationship between binding affinity and evaluated function of ABC transporter subfamily molecules to Cry toxins. Citations: a, [34]; b, [36]; c, [41]; d, [42]; e, [35]. Susceptibility-determining activity was determined using knockout *B*. *mori* larvae, and receptor function was evaluated by cell swelling assays using heterologous expression systems of Sf9 cells (**A**) or both Sf9 and HEK293T cells (**B**).

**Figure 6:**
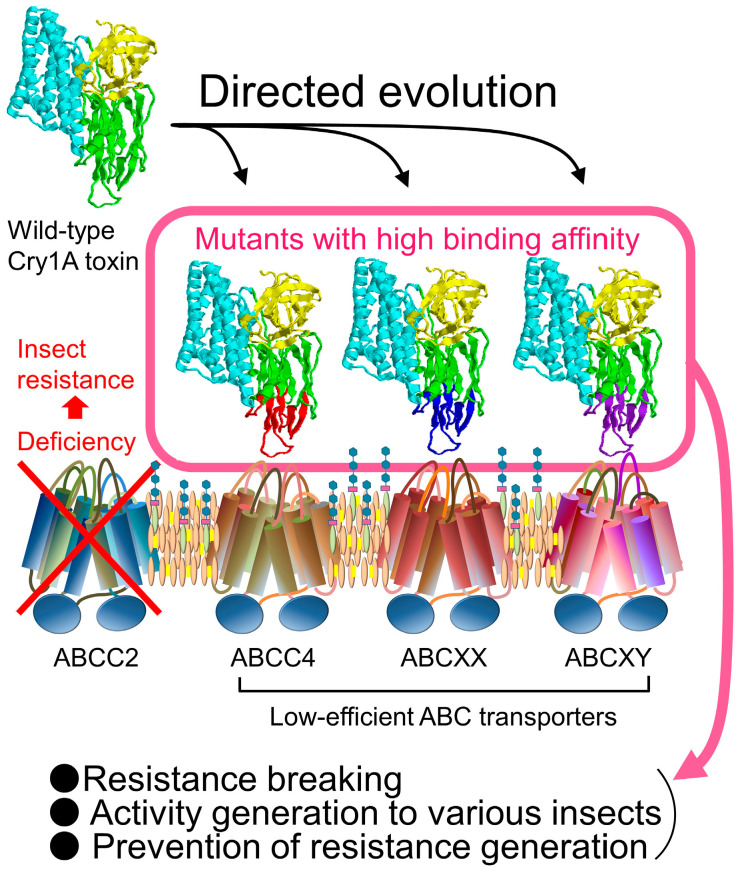
Schematic diagram of an application example of directed evolution of Cry1A toxin targeting ABC transporters as low-efficiency receptors.

Since 2017, several ABC transporters have been identified as low-efficiency receptors that poorly induce cell swelling when heterologously expressed in cultured cells [34,36,42]. By 2024, a range of ABC transporter subfamily molecules, including ABCC1, ABCC2, ABCC3, ABCC4, ABCC10, and ABCC11, were discovered in the midgut of silkworm larvae as low-efficiency receptors to Cry toxins, which do not play a role in determining susceptibility [41] (Figure 5; Table 1). These findings are considered significant for understanding the evolutionary trajectory of each Cry toxin subfamily. Moreover, this discovery opens new possibilities for the molecular engineering of Cry toxins, especially in the context of the increasing difficulty of discovering new Cry toxins and the development of pest resistance through the deletion of ABC transporter genes. Therefore, as the third topic of this review, an effort was made to compile information on low-efficiency receptors, despite the limited number of reports available. Finally, this review attempts to present some strategies for the engineering of Cry toxins, drawing lessons from the presumed evolutionary mechanisms of Cry toxins (Figure 6).

## 2. Models of Receptors Involved in Determining Susceptibility to Cry Toxin in the Silkworm

The roles of ABC transporters and Cad in determining the susceptibility of individual insects, particularly in the silkworm, have been systematically analyzed since 2016 through heterologous expression systems, knockout by genome editing, and binding affinity analysis using surface plasmon resonance (SPR). This section reviews the results of these analyses and attempts to diagram several models of typical receptor usage (Figure 1).

### 2.1. BmABCC2, BmABCC3, and BmCad Usage Model for Cry1Aa Toxin

In a toxin overlay assay with Cry1Aa using knockout silkworms, knockout of BmCad resulted in ~100 times more resistance [35]. This finding suggests the loss of cooperative and synergistic effects created by the combination of BmCad with BmABCC2 and BmCad with BmABCC3. Conversely, knocking out BmABCC2 or BmABCC3 alone did not confer resistance, but simultaneous knockout of both resulted in over 4100 times resistance [35]. This indicates that the cooperative and synergistic ion channel formation activities produced by combinations of BmCad and BmABCC2 and of BmCad and BmABCC3 are equivalent and crucial for determining the susceptibility of *B*. *mori* larvae to Cry1Aa toxin [21,35] (Figure 2A). In the heterologous expression system of Sf9 cells, BmABCC2 exhibited 100 times higher cell-swelling-inducing activity against Cry1Aa than BmABCC3 as a single molecule. However, the synergistic effect achieved with BmCad and BmABCC3 exerted the same level of synergistic activity as BmCad and BmABCC2 [35]. BmABCC3 has a low binding affinity (*KD* = 3.4 × 10^−8^ M) for Cry1Aa, while BmCad has a high binding affinity (*KD* = 7.2 × 10^−10^ M) [38]. BmABCC3, in cooperation with BmCad, might exert a nearly 1000 times greater synergistic effect by efficiently collecting Cry1Aa from the concentration range of 10^−10^ M, a range in which Cry1Aa cannot be collected by BmABCC3 alone [35]. In contrast, BmABCC2 has a high binding affinity for Cry1Aa (*KD* = 3.1 × 10^−10^ M) [43], and since BmABCC2 can effectively collect Cry1Aa by itself, the effect of BmCad in collecting Cry1Aa may be relatively lower than that observed with BmABCC3.

In the Sf9 heterologous expression system, BmABCC4 exhibited cell-swelling-inducing activity that was 1000 times lower than that of BmABCC2 [41]. In addition, in experiments using a knockout strain of silkworms, double knockout of BmABCC2 and BmABCC3 reduced susceptibility to Cry1Aa by more than 10,000 times. Therefore, in silkworm larvae, BmABCC4 is considered to confer more than 10,000 times lower susceptibility than BmABCC2 and BmABCC3 to Cry1Aa. In fact, knocking out BmABCC4 did not result in resistance to Cry1Aa [41]. These indicated that high expression level of BmABCC4 in the midgut does not contribute to susceptibility due to its low binding affinity for Cry1Aa (*KD* = 2.7 × 10^−7^ M) [41] (Figure 2A and Figure 5B; Table 1).

Synergism is believed to occur when two types of molecules function differently. BmABCC2 demonstrates a significantly higher ion-channel-formation-promoting activity than BmCad, leading to increased cell-swelling-inducing activity [17,21]. It has been reported that human ABCC exists in lipid rafts, which are characterized by a high melting point and low fluidity [44], and it is presumed this is also the case in insects. Additionally, in experiments using brush border membrane vesicles from the midgut of *H*. *virescens*, Cry1Ac toxin was found to accumulate in the Triton-insoluble compartment, believed to be a lipid raft [45]. These findings suggest that BmABCC2 facilitates membrane penetration of Cry toxin. Conversely, Cad has been reported to induce proteolytic activation of the toxin, where helix α-1 is cleaved in monomeric Cry1A toxin, resulting in the exposure of hydrophobic residues [46]. This conformational change is thought to further facilitate the formation of a pre-pore oligomeric structure. Moreover, Cad is found in highly fluid phospholipids [47], and the quantity of BmCad molecules expressed in the midgut of silkworm larvae was ~2.5 times that of BmABCC2 [34]. Therefore, Cad may have a synergistic relationship with ABCC by effectively delivering Cry toxin or its pre-pore structure to the less mobile ABCC (Figure 2A). Additionally, the function of inducing programmed cell death via signal transduction was also considered a potential role for Cad in synergism [48,49]. However, in the heterologous expression system of Sf9, even a mutant BmCad that possessed only the extracellular domain demonstrated synergism with BmABCC2 in cell-swelling induction [42]. This suggests that the role of Cad required for synergism is not the induction of programmed cell death via signal transduction.

### 2.2. BmABCC2 and BmCad Usage Model for Cry1Ac Toxin

BmABCC2 has been confirmed to act as a receptor for Cry1Ac toxin in heterologous expression systems of Sf9 cells and *Xenopus* oocytes [17,21]. Additionally, in heterologously expressed cultured cells, ABCC3 from both *Spodoptera litura* and *B*. *mori* facilitated cell swelling corresponding to Cry1Ac intoxication [36,42,50]. However, the role of ABCC3 in determining the susceptibility of individual insects to Cry1Ac toxin varied. In *Plutella xylostella*, resistance to Cry1Ac was observed by knocking out either ABCC2 or ABCC3, with even higher resistance manifested when these two ABCCs were simultaneously knocked out [31], a phenomenon that challenges the principle that receptors with higher functionality determine susceptibility. Conversely, according to an experiment by Zhao et al. (2021) [33], resistance to Cry1Ac in *P*. *xylostella* emerged only upon double knockout of ABCC2 and ABCC3. Similarly, Cry1Ac resistance in *H*. *armigera* was observed only when both ABCC2 and ABCC3 were knocked out [32]. In these instances, ABCC2 and ABCC3 are thought to function equivalently.

In the silkworm, BmABCC2 has been identified as a key molecule determining sensitivity to Cry1Ac through mutation introduction using genome editing [10]. Its mechanism, including its interaction with Cad, has been thoroughly analyzed. Knockout of BmABCC2 led to more than a 250-fold increase in resistance to Cry1Ac [34]. A knockout of BmCad also resulted in a more than 10-fold increase in resistance, while knockout of BmABCC3 had no significant effect [34]. In a heterologous expression system using Sf9 cells, BmCad displayed cooperative and synergistic cell-swelling-inducing activity with BmABCC2 against Cry1Ac [34]. These findings suggest that the synergism between BmABCC2 and BmCad determines the sensitivity to Cry1Ac in silkworm larvae (Figure 2B). This also indicates that the receptors used by Cry1Ac in *P*. *xylostella* and *H*. *armigera*, as previously mentioned [32,33], differ from those in *B*. *mori*, which is expected given that each ABC transporter molecule from different insects likely possesses distinct binding characteristics for the same Cry toxin.

In the heterologous expression system of Sf9, BmCad was shown to act synergistically with not only BmABCC2 but also BmABCC3 against Cry1Ac [34]. However, the synergistic receptor activity between BmCad and BmABCC2 was found to be over 1000 times higher than that between BmCad and BmABCC3. Therefore, it is believed that due to this reduced synergistic receptor activity, BmABCC3 does not play a significant role in the susceptibility of *B*. *mori* larvae, leading to resistance appearing when only BmABCC2 or BmCad is knocked out (Figure 2B). Although the binding affinity of BmABCC3 for Cry1Ac (*KD* = 8.1 × 10^−8^) is only slightly less than its affinity for Cry1Aa (*KD* = 3.4 × 10^−8^) (Figure 2A,B), the receptor function through synergism is considerably reduced concerning Cry1Ac [34]. The reason for this discrepancy remains unclear. However, knockout of BmABCC2 alone resulted in a greater than 3200-fold increase in resistance, indicating that BmABCC3 exhibits almost no function in synergy with BmCad. It is possible that factors beyond binding affinity contribute to receptor activity or synergism.

A model of receptors for Cry1Ab is not discussed here. However, in both the silkworm larval system and Sf9 heterologous expression systems, it has been demonstrated that the receptors determining sensitivity to Cry1Ab and Cry1Ac are very similar [34] (Figure 1).

### 2.3. BmABCC2 and BmABCC3 Usage Model for Cry1Fa Toxin

In the case of Cry1Fa, no synergism was observed between BmABCC2, BmABCC3, and BmCad in the heterologous expression system of Sf9 cells [34]. Furthermore, there was no difference in susceptibility to Cry1Fa between BmCad knockout silkworm larvae and the wild type, suggesting that BmCad does not play a functional role in the sensitivity of individual silkworm larvae to Cry1Fa (Figure 3A). This lack of function is attributed to the low binding affinity of Cry1Fa for BmCad (*KD* = 4.0 × 10^−9^ M), which is lower than its binding affinity for BmABCC2 and BmABCC3 (Figure 3A). Additionally, in silkworm larvae, knocking out either BmABCC2 or BmABCC3 alone did not affect resistance, but simultaneous knockout of both BmABCC2 and BmABCC3 resulted in an increase in resistance of more than 100-fold [34]. This outcome indicates that BmABCC2 and BmABCC3 contribute to equivalent levels of susceptibility in silkworm larvae (Figure 3A). However, the dissociation constants for Cry1Fa with BmABCC2 and BmABCC3 were 2.02 × 10^−10^ M and 2.85 × 10^−9^ M, respectively, suggesting that the former has a 10 times higher binding affinity than the latter. Moreover, BmABCC2’s cell-swelling-inducing function in the Sf9 heterologous expression system was 10 times greater than that of BmABCC3 [34]. Therefore, a discrepancy exists between the relationship of binding affinity and receptor function in a heterologous expression system and the actual susceptibility conferred in silkworm larvae. As Endo et al. (2022) [38] previously suggested, factors beyond binding affinity may contribute to the susceptibility conferred by BmABCC3 in individual silkworm larvae.

As discussed, in determining the susceptibility of silkworm larvae, three molecules (BmABCC2, BmABCC3, and BmCad) are involved in Cry1Aa intoxication, two molecules (BmABCC2 and BmCad) in Cry1Ac intoxication, and two molecules (BmABCC2 and BmABCC3) in Cry1Fa intoxication. However, as noted in the section on the Cry1Ac receptor model, the binding affinities of ABCC2, ABCC3, and Cad to Cry toxins may vary among insects, even for the same Cry toxin. Consequently, the receptors used by Cry1A toxins might differ for each insect species.

### 2.4. BmABCB1 Usage Model for Cry1Ia Toxin

No difference in susceptibility to Cry1Ia was observed between BmCad knockout and wild-type silkworm larvae, indicating that BmCad does not play a functional role in the response of individual silkworm larvae to Cry1Ia [29]. This lack of function is attributed to the low binding affinity between Cry1Ia and BmCad (*KD* = 3.1 × 10^−8^ M) (Figure 3B). Conversely, silkworm larvae with a knockout of BmABCB1 exhibited resistance exceeding 10,000-fold. Knockouts of BmABCC2, BmABCC3, and BmCad, which are receptors for Cry1Aa, did not demonstrate any resistance [29]. Therefore, among the nine subfamilies of insect ABC transporters [51], Cry1Ia appears to specifically interact with the ABCB subfamily, which is phylogenetically adjacent to the ABCC subfamily [29] (Figure 1 and Figure 3B).

Additionally, BmABCB1 knockout silkworm larvae showed over 100 times resistance to Cry1Ba and Cry9Da [29]. In the leaf beetle *Chrysomela tremula*, resistance to Cry3Aa was attributed to the disruption of CtABCB1, with cell-swelling tests in CtABCB1-expressing Sf9 cells confirming its function as a Cry3Aa receptor [26]. In the western corn rootworm, *Diabrotica virgifera virgifera*, knockdown of DvABCB1 by RNA intereference rendered the larvae insensitive to a Cry3A toxin. Heterologous expression of DvABCB1 in Sf9 and HEK293 cells conferred sensitivity to the cytotoxic effects of Cry3A toxins [27]. Therefore, ABCB1 is considered a common receptor determining insect susceptibility to these Cry toxins. Despite the phylogenetic distance between Cry1Ia, Cry1Ba, Cry9Da, and Cry3Aa in a tree based on the entire activated toxin’s amino acid sequence [52], they cluster in the same clade in a tree based on the amino acid sequence of domain II [53] (Figure 1). This suggests that the receptor-binding site for these four Cry toxins is located in domain II, with high sequence similarity within this domain.

### 2.5. BmABCA2 Usage Model for Cry2Aa and Cry2Ab

Knocking out BmABCA2 resulted in 100-fold resistance to Cry2Aa and 10,000-fold resistance to Cry2Ab, indicating BmABCA2 as a key determinant of susceptibility to these toxins [28]. BmABCA2-expressing HEK292T cells exhibited cell-swelling-inducing activity at 40 nM of these toxins. These suggest the critical role of BmABCA2 in the susceptibility of silkworm larvae to Cry2Aa and Cry2Ab (Figure 3C). The effect of BmCad knockout on susceptibility to Cry2Aa and Cry2Ab has not been reported, leaving BmCad’s involvement in Cry2A susceptibility unknown. Knockdown of two types of Cad in the striped rice stem borer, *Chilo suppressalis*, resulted in mild resistance to Cry2A [54], but the equal resistance produced by knockdown of both Cad types challenges the principle, questioning the conclusion that they function as receptors for Cry2A toxin. Furthermore, *H*. *armigera* Cad expressed in Sf9 cells did not induce swelling against Cry2Ab, and silencing Cad in *H*. *armigera* larvae did not generate resistance to Cry2Ab, suggesting Cad’s non-functionality in the response of *H*. *armigera* to Cry2Ab [30].

## 3. Receptor-Binding Site Created by the Domain II Loops of Cry Toxin and Its Promiscuous Properties

The Structural Classification of Proteins database (SCOP; https://scop.mrc-lmb.cam.ac.uk/ accessed on 26 March 2024) classifies domain II of the Cry toxin as the beta-prism I fold, similar to the structure found in *Maclura pomifera* agglutinin, Jacalin, which is known for its lectin function binding to galactose (Gal) and methyl-α-N-acetyl galactosamine (Me-α-GalNAc) [55,56]. Similarly, the cavity of beta-prism I fold of banana lectin (BanLec) binds to various sugars like laminaribiose (Glcβ1-3Glc) and Xyl-β1,3-Man-α-O-Methyl [57]. These suggest an evolutionary path of the cavity of beta-prism I fold towards acquiring binding properties to sugar chains. However, the evolutionary trajectory of cavities is not limited to sugar chain interactions. Cavities of these lectins are analogous to the cavity formed by loops α8, 1, 2, and 3 in domain II of the Cry toxin (Figure 4). As we will discuss further, the cavity created by loops of the Cry toxin might have evolved to bind to a wide array of proteins, including various ABC transporter subfamilies and Cad.

The binding of Cry1Aa to *Manduca sexta* Cad, BtR1, was inhibited by synthetic loops 2 and 3 of Cry1Aa, and similarly, binding to Cry1Ab was blocked by synthetic peptides corresponding to loops α8 and 2 [58,59]. Furthermore, binding of Cry1Ac to *H*. *virescens* Cad, HevCaLP, was inhibited by a synthetic peptide corresponding to loop 3 [60]. Hence, the binding site for Cad on Cry1A toxins is identified in the region formed by loops α8, 2, and 3.

Further analysis on the Cad binding region of Cry1Aa focused on BmCad from the silkworm. Epitope mapping with a monoclonal antibody that blocks binding of Cry1Aa to BmCad identified the binding site within a loop region [61]. Inhibition experiments using cross-linking tags, such as 5-iodoacetamidofluorescein or N-(9-acridinyl) maleimide, pinpointed R311 in loop 1, N376 in loop 2, and G442 and Y445 in loop 3 as critical for BmCad binding [61,62]. Deletion of consecutive amino acids in loop 1 significantly reduced cell-swelling activity in BmCad-expressing Sf9 cells [43]. Additionally, cross-linking mutations that altered the structure or closed the cavity entrance, specifically mutants bridging loops α8 and 2 (R281-G442, R281-I369), decreased the binding affinity to BmCad [37]. Thus, it is suggested that the Cad binding region on Cry1A toxins encompasses a broad area of the cavity formed by loops α8, 1, 2, and 3 (Table 1), which gives the characteristics of susceptibility-determining receptors, low-functional receptors, and non-functional receptors.

In dot-blot assays on Sf9 cell membranes expressing BmABCC2, Cry1Aa binding was significantly inhibited by BmCad, indicating a competitive relationship between the binding sites of BmABCC2 and BmCad on Cry1Aa [43] (Figure 4). Despite this, cross-linked mutants in loops α8, 2, and 3, which substantially decreased binding affinity to BmABCC2, retained some binding affinity to BmCad, though reduced [37]. Additionally, single amino acid residue substitution experiments on Cry1Aa revealed that mutants including Y366V, R367D, and R368D significantly impacted binding to BmABCC2 without affecting binding to BmCad. Thus, although direct confirmation of receptor molecules binding within the cavity by X-ray crystallography is pending, the binding site is presumed to be extensive and varies depending on the target. This characteristic of the cavity’s binding was described as “promiscuous” [37] (Figure 4).

As noted in Section 2, Cry1Ia, Cry1Ba, Cry9Da, and Cry3Aa, which share similar domain II structures, all use ABC transporter B1 as a receptor, suggesting the potential of the Cry toxin’s promiscuous binding cavity to engage with different ABC transporter subfamilies [26,27,29]. This promiscuous nature might also explain the existence of ABC transporters with low binding affinity that do not contribute to the susceptibility of individual insects, termed low-efficiency receptors [41] (see Section 4). Moreover, this characteristic may underlie the variability in the use of Cad by different Cry toxin subfamilies [34] (Figure 1). However, these concepts remain to be conclusively demonstrated, and further reliable data are anticipated.

The receptor for Cry5, which is lethal to nematodes, is a glycolipid known as Component B, with binding epitopes including galactose and GalNAc [39,40]. Although direct evidence linking domain II as the binding site for glycolipids is yet to be established, the β-prism I fold of domain II and the evolution of similar structures as lectins, such as Jacalin and BanLec [56,57], suggest the possibility that the glycolipid binding site of Cry5 is located within the cavity formed by loops of domain II.

## 4. Roles of ABC Transporters as Low-Efficiency Receptors and Inefficient Receptors to Cry Toxins

Using the transient expression system in HEK293T cells, receptors to Cry toxins were investigated among six subfamilies of BmABCC molecules expressed in the midgut of silkworm larvae [41]. In this study, beyond the already recognized BmABCC2 and BmABCC3, which are associated with susceptibility, BmABCC4 was identified to induce cell swelling at a high Cry1Aa concentration of 1700 nM. Similarly, Cry1Ca, BmABCC1 and BmABCC10; Cry1Da, BmABCC4, BmABCC10, and BmABCC11; Cry8Ca, BmABCC2, and BmABCC4; and Cry9Aa, BmABCC1, BmABCC3, BmABCC10, and BmABCC11 were found to facilitate cell swelling, albeit at toxin concentrations exceeding 1000 nM. This suggests the presence of various ABCC molecules in the silkworm midgut capable of inducing cell swelling in HEK293T cells expressing these molecules at high Cry toxin concentrations, albeit with low activity.

Further examination of cell-swelling-inducing activity against Cry1Aa, Cry1Da, Cry8Ca, and Cry9Da used the Sf9/Baculovirus heterologous expression system for more quantitative and reproducible results. Cells expressing BmABCC1 initiated cell swelling at 500 nM of Cry9Da toxin. Additionally, BmABCC4-expressing cells began to swell upon administration of 5 μM of Cry1Aa, Cry1Da, and Cry8Ca toxins. These concentrations are significantly higher (5000 and 50,000 times, respectively) than the concentration of Cry1Aa causing cell swelling in Sf9 cells expressing BmABCC2, the receptor determining susceptibility to Cry1Aa. The binding affinities of these toxins to their respective receptors were in the range of 10^−7^ M to 10^−9^ M, as expressed by *KD* values [41], which are 10 to 1000 times higher than the *KD* values observed between Cry1Aa, Cry1Ab, Cry1Ac, and their susceptibility-determining receptor BmABCC2 [34,36] (Table 1). Moreover, knockout of BmABCC1 or BmABCC4 did not affect the susceptibility of silkworm larvae to Cry1Aa, Cry1Da, Cry8Ca, and Cry9Da, indicating that BmABCC1 and BmABCC4 function as low-efficiency receptors for Cry9Da, and Cry1Aa, Cry1Da, and Cry8Ca, respectively, but do not act as determinants of susceptibility in individuals. Therefore, these ABCCs were categorized as low-efficiency receptors [41]. Additionally, many ABC transporter subfamily molecules present in the midgut are considered inefficient for the Cry toxin subfamily (Table 1; Figure 5A).

The discovery that there are 1–4 types of low-efficiency ABCCs for each Cry toxin subfamily emerged from a small-scale screening involving only six types of ABCCs (Figure 5A). It was reported that 45 types of ABC transporter subfamilies are expressed in the silkworm midgut [41], suggesting the potential existence of approximately seven low-efficiency receptors for each Cry toxin among these transporters. However, expression levels vary among the 45 types of ABC transporters in silkworm larvae’s midgut, with 19 having expression levels comparable to BmABCC2 and BmABCC3, which are known to function as highly efficient susceptibility-determining receptors [41]. Consequently, it is conceivable that 3 to 12 types of ABC transporters may act as low-efficiency receptors with high expression levels for each Cry toxin subfamily in silkworm larvae. This implies that, in actual insect midguts, Cry toxins could potentially engage a surprisingly large number of ABC transporters as low-efficiency receptors. Although these receptors have low binding affinity for Cry toxins and do not contribute to the toxicity of *Bt* to individual insects, mutants of Cry toxins with enhanced binding affinity for these low-efficiency receptors could lead to the selection of *Bt* strains capable of killing insects and obtaining nutrients. This suggests a possible evolutionary pathway for *Bt* to kill various insects, leveraging the promiscuous properties of the Cry toxin receptor-binding cavity (Figure 4). Currently, this remains a hypothesis, awaiting further evidence.

Some ABCC molecules have also been identified that align with the concept of low-efficiency receptors or inefficient receptors for other Cry toxins. The *KD* values for the interactions between Cry1Ab and BmABCC3 and between Cry1Ac and BmABCC3 are 4.35 × 10^−8^ M and 8.13 × 10^−8^ M, respectively (Table 1). These *KD* values are ~10 and 20 times higher than those for interactions between Cry1Ab and BmABCC2 and Cry1Ac and BmABCC2, respectively [34]. In terms of sensitivity, Sf9 cells heterologously expressing BmABCC3 showed ~500 times lower sensitivity to Cry1Ab than cells expressing BmABCC2 (Table 1). Moreover, BmABCC2 knockout silkworm larvae exhibited over 1000 times resistance to Cry1Ab and Cry1Ac, whereas BmABCC3 knockout larvae showed the same susceptibility as wild-type larvae and did not develop resistance [34]. These indicate that BmABCC3 does not act as a determinant of susceptibility to Cry1Ab and Cry1Ac, categorizing it as a low-efficiency receptor with low binding affinity for these toxins. The *KD* value for the interactions between Cry1Ca and BmABCC2 and between Cry1Ca and BmABCC3 are 1.71 × 10^−7^ M (Table 1) and 3.92 × 10^−7^ M, respectively [36]. The *KD* value for the interactions between Cry1Da and BmABCC2 and between Cry1Da and BmABCC3 are 2.30 × 10^−6^ M (Table 1) and 4.18 × 10^−4^ M, respectively [36]. Both Cry1Ca and Cry1Da were not active against either Sf9 cells heterologously expressing BmABCC2 (Table 1) or BmABCC3. Although both receptors have low binding affinity to these toxins, these suggest that both BmABCC2 and BmABCC3 are inefficient receptors for Cry1Ca and Cry1Da. Furthermore, Sf9 cells heterologously expressing BmABCC2 and BmCad showed low sensitivity to Cry8Ca [17]. Although there is no report on the *KD* values for the interactions between Cry8Ca and BmABCC2, this suggests that BmABCC2 is a low-efficiency receptor for Cry8Ca in the midgut of silkworm larvae.

The *KD* value for the interactions between coleopteran specific Cry8Ca and *Tribolium castaneum* ABCC4 (TcABCC4) is 4.03 × 10^−8^ M [42] (Table 1). HEK293T cells heterologously expressing TcABCC4 showed low sensitivity to Cry8Ca (Table 1). In addition, *although* the midgut of *T. castaneum* larvae expresses TcABCC4, the larvae survived for two weeks in a bioassay with a high concentration of Cry8Ca toxins (1.2 mg/g diet) [42]. These suggest that TcABCC4 is a low-efficiency receptor for Cry8Ca (Figure 5B). In contrast, the *KD* value for the interactions between Cry3Ba and TcABCC4 is 1.78 × 10^−5^ M (Table 1). But HEK293T cells heterologously expressing TcABCC4 did not show sensitivity to even 1.7 μM Cry8Ca (Table 1). These suggest that TcABCC4 is an inefficient receptor for Cry3Ba (Table 1).

It has been demonstrated that a correlation exists between the binding affinity of ABCC transporters and their capacity to induce cell swelling in response to Cry1Aa toxin [34,35,38,43]. Table 1 summarizes the features of susceptibility-determining receptors, low-efficiency receptors, and inefficient receptors, covering aspects such as binding affinity, susceptibility in heterologously expressed cells, and their contribution to the susceptibility of individual insects. When the *KD* between an ABCC and a Cry toxin is in the range of 10^−10^ M, Sf9 cells expressing the ABCC demonstrate high sensitivity, showing swelling at toxin concentrations from 100 pM to 10 nM [17,36,38,43] (Table 1; Figure 5B). These ABCC transporters with high cell-swelling-inducing activity are crucial for conferring susceptibility to individual insects, which exhibit high resistance upon their knockout [34,35] (Table 1). However, when two ABCC molecules possess the same high level of functionality (as described in Section 2 with the relationship between BmABCC2 and BmABCC3 against Cry1Aa and Cry1Fa), both molecules contribute to susceptibility, and insect individuals do not exhibit resistance when only one of the ABCC molecules is knocked out [34,35].

At a *KD* of 10^−9^ M between ABCC and Cry toxin, Sf9 cells expressing some ABCC molecules start to swell at toxin concentrations ranging from 10 to 500 nM [34,41] (Table 1). The role of ABCCs in conferring susceptibility to individual insects varies, with BmABCC3 functioning for Cry1Fa but not BmABCC1 for Cry9Aa, indicating that at this level, ABCCs may act as borderline functional receptors (Figure 5B).

With a *KD* of 10^−8^ M between ABCC and Cry toxin, the functionality of ABCCs becomes more varied. Some ABCCs with a *KD* at this level exhibit high cell-swelling activity in Sf9 cells and serve as susceptibility-determining receptors for individual insects [29,41]. However, many ABCCs at this *KD* level either induce weak cell-swelling activity in Sf9 cells or are undetectable, failing to act as susceptibility-determining receptors for individual insects (Table 1; Figure 5B).

When the *KD* between ABCC and Cry toxin exceeds 10^−7^ M, the cell-swelling activity of ABCCs in Sf9 cells tends to be absent, and there are no instances where ABCCs act as susceptibility-determining receptors for individual insects [41] (Table 1; Figure 5B). The *KD* values of 4.61 × 10^−5^ M for BmABCC1 against Cry1Aa and 1.78 × 10^−5^ M for TcABCC4 against Cry3Bb, obtained through precise SPR method sensorgrams [36,41], suggest the presence of many molecules in the midgut acting as inefficient receptors despite such binding affinities to Cry toxins.

## 5. Protein Engineering of Cry Toxin by Imitating the Mechanism of Evolution

As mentioned above, Cry toxin is believed to interact with a large number of low-efficiency receptors in the insect midgut (Section 4), influenced by the promiscuous nature of the binding cavity (Section 3). Additionally, Cry toxins are thought to have adapted to increase their binding affinity for certain molecules within these low-efficiency receptors (Section 4) and have evolved to use these molecules as susceptibility determinants, leading to the development of toxicity against a broad range of insects (Section 2). Directed evolution represents a fitting molecular engineering approach to enhance binding affinity. Thus, this method could replicate the evolutionary pattern discussed here to enhance or develop new Cry toxins.

### 5.1. Directed Evolution of Cry Toxin Targeting Cad

Directed evolution efforts have primarily focused on Cry1Aa toxin, targeting BmCad [63,64]. These endeavors led to a 42-fold and 50-fold increase in the binding affinity of Cry1Aa to BmCad through sequential substitution of four amino acids in loop 3 (439QAAG442) and loop 2 (371LGSG374), respectively [64,65]. Moreover, a combination of mutations in loops 2 and 3 resulted in a 50-fold enhancement in binding affinity to BmCad [66]. Despite these advances, the insecticidal activity against silkworm larvae did not improve in any instance. As discussed earlier, the original binding affinity of BmCad for Cry1Aa, with a *KD* value of 7.2 × 10^−10^ M, is not significantly different from the *KD* value of 3.1 × 10^−10^ M between BmABCC2 and Cry1Aa [43]. Furthermore, the *KD* value between BmCad and Cry1Aa is much higher than that between BmABCC3 and Cry1Aa, which is 3.4 × 10^−8^ M [36]. Therefore, if the synergistic function of BmCad involves delivering oligomerized Cry1Aa to ABCC molecules, as hypothesized, the increased binding affinity of Cry1Aa mutants to BmCad may exceed what is necessary for synergism and thus may not enhance the insecticidal efficacy of the toxin.

Badran et al. (2016) [67] advanced the directed evolution of Cry1Ac toxin targeting *T*. *ni* Cad (TnCad) through a method called phage-assisted continuous evolution (PACE). Initially, Cry1Ac lacked confirmed binding affinity for TnCad, but mutations introduced via PACE enabled the mutant to acquire binding affinity to TnCad and exhibit weak cell-swelling activity in Sf9 cells expressing TnCad [67]. This achievement likely stems from targeting Cad, which naturally has a low binding affinity for Cry1Ac toxin. Despite this, no notable increase in insecticidal activity was observed against wild-type *T*. *ni* larvae. Given the previously demonstrated minimal ion-channel-formation-inducing activity of BmCad [21], TnCad might significantly influence *T*. *ni* larvae susceptibility through cooperative and synergistic action with TnABCC2. The limited insecticidal activity of the Cry1Ac mutant against *T*. *ni* larvae could be due to its binding affinity for TnCad (*KD* = 1.1 × 10^−8^ M) not being sufficient to facilitate cooperative and synergistic action with TnABCC2.

Furthermore, BmCad knockout silkworm larvae displayed 100 times resistance to Cry1Aa [35], 250 times resistance to Cry1Ab, and 10 times resistance to Cry1Ac [34]. This indicates the role of Cad in susceptibility through enhancing the ion channel formation activity of ABC transporters. Therefore, directed evolution of Cad should aim to boost its cooperative and synergistic activity with ABC transporters, particularly when the binding affinity between the toxin and cadherin is low, as seen with Cry1Fa and BmCad (Table 1; Figure 3A).

The success of directed evolution greatly depends on the mutation sites. For PACE selection targeting TnCad, mutations were introduced at random Cry1Ac sites [67]. The effectiveness of this seemingly inefficient random method can be attributed to PACE’s robust combination of mutation introduction and selection. Conversely, the selection of Cry1Aa mutants aimed at BmCad was performed using more traditional methods, such as phage display and panning. Despite the mutant library’s diversity being limited to the order of 10^5^, mutants exhibiting 42-, 50-, and 50-fold increases in binding affinities were identified [64,65,66]. This high efficiency in selection is believed to result from focusing mutation introduction on the loops of domain II, which serve as the Cad binding site. Typically, achieving significantly improved binding affinity through random mutations requires multiple rounds of mutation introduction and selection. However, targeting mutations specifically to the loop region of domain II resulted in nearly 50-fold enhanced binding affinity to Cad with only a single mutation [64,65], highlighting the strategic significance of targeted mutation locations.

### 5.2. Directed Evolution of Cry Toxin Targeting ABC Transporters

As hypothesized in Section 4, Cry toxins may achieve insecticidal efficacy by binding with high affinity to ABC transporters that act as low-efficiency receptors, potentially reflecting an evolutionary pathway Cry toxins have undergone naturally. Directed evolution could thus mimic these natural evolutionary principles to develop Cry toxins.

There are three potential uses for the directed evolution of Cry toxin targeting ABC transporters. The first is breaking resistance. Resistance has emerged in many insect pests due to ABC transporter deficiencies, leading to reduced effectiveness of genetically modified *Bt* crops. This resistance could be countered by incorporating a mutant toxin with high binding affinity for the low-efficiency receptors of the ABC transporter in the insect midgut (Figure 6). Using a Cry toxin already deployed in crops could be more beneficial since the safety data for that toxin are readily available. The second application involves creating mutant toxins effective against pests that currently lack potent Cry toxin countermeasures. Rather than screening for natural Cry toxins, mutants developed through directed evolution could be employed. The third application builds on the first two, aiming to generate multiple Cry toxin mutants for various low-efficiency ABC transporters for concurrent use (Figure 6). Introducing several mutant toxins into crops simultaneously could prevent pests from easily developing resistance to a single Cry toxin type. This strategy is already in use in genetically modified *Bt* crops featuring multiple genes. Despite the limited variety of Cry or *Bt* toxins available for use, directed evolution could facilitate the creation of four or five effective mutants for simultaneous crop introduction. Developing mutant toxins with enhanced insecticidal activity through directed evolution holds promise not only for genetically modified *Bt* crops but also for creating new *Bt* spray formulations.

## 6. Conclusions

Research has shown that the receptors involved in determining susceptibility vary according to the Cry toxin subclass, illustrating a complex interaction network where Cry toxins engage divergent ABC transporter subfamilies and Cad. Additionally, numerous ABCCs in the insect midgut have been identified with low efficiency in inducing toxicity, rather than a susceptibility-determining function against insects. It has also been shown that the loop region’s cavity site serves as a common binding site for Cry1Aa to ABCC2 and Cad, exhibiting “promiscuous property” which enables it to bind various receptors. These have opened up new perspectives on the evolutionary trajectory of Cry toxins, suggesting that the evolution of Cry toxins may be driven by their ability to bind various ABCCs, Cad, and sugar chains through their promiscuous binding cavity.

## Figures and Tables

**Table 1:** Characteristics of susceptibility-determining receptors, low-efficient receptors and inefficient receptors.

Function of Receptor	Receptor	Cry Toxin	*KD* between Receptor and Cry (M)	Swelling Starting Toxin Conc of Heterologously Receptor-Expressing Sf9 (nM)	Susceptibility of KO *B. mori* Larvae	Reference
Low-efficiency or inefficient	BmABCC1	Cry1Aa	4.61 × 10^−5^	>5000	ND	[41]
		Cry1Da	4.15 × 10^−7^			
		Cry8Ca	3.48 × 10^−8^			
		Cry9Aa	1.89 × 10^−9^	500	Susceptible	
	BmABCC2	Cry1Ca	1.71 × 10^−7^	ND ^1^	ND	[36]
		Cry1Da	2.30 × 10^−6^			
		Cry3Bb	1.96 × 10^−5^			
	BmABCC3	Cry1Ab	4.35 × 10^−8^	1000	Susceptible	[34]
		Cry1Ac	8.13 × 10^−8^	>1000		
	BmABCC4	Cry1Aa	2.67 × 10^−7^	5000		[41]
		Cry1Da	6.37 × 10^−8^			
		Cry8Ca	6.17 × 10^−8^			
		Cry9Aa	ND	>5000	ND	
	TcABCC4	Cry8Ca	4.03 × 10^−8^	ND ^2^		[42]
		Cry3Bb	1.78 × 10^−5^	ND ^1^		
	BtR175	Cry1Fa	3.95 × 10^−9^	>1000		[34]
		Cry1Ia	3.09 × 10^−8^ M	ND		[29]
Susceptibility determining	BmABCC2	Cry1Aa	4.30 × 10^−10^	0.1	Susceptible	[17,35,36]
		Cry1Ab	2.57 × 10^−10^	10	Resistant	[34]
		Cry1Ac	2.34 × 10^−10^	1		
		Cry1Fa	2.02 × 10^−10^	1	Susceptible	
	BmABCC3	Cry1Aa	3.42 × 10^−8^	10		[34,35,36]
		Cry1Fa	2.85 × 10^−9^	10		
	BtR175	Cry1Aa	7.2 × 10^−10^	1000	Resistant	[35,43]
		Cry1Ab	5.49 × 10^−10^	1000		[34]
		Cry1Ac	2.97 × 10^−10^	>1000		

ND, not determined. ^1^, not toxic using 1 μM toxin in HEK293T assay. ^2^, toxic using 1 μM toxin in HEK293T assay.

## Abbreviations

ABC transporters, ATP-binding cassette transporters; ABCC, ABC transporter subfamily C; ABCB, ABC transporter subfamily B; ABCA, ABC transporter subfamily A; Cad, cadherin-like receptor; *KD*, dissociation constant.
